# HeartLogic multisensor algorithm response prior to ventricular arrhythmia events

**DOI:** 10.1002/joa3.12913

**Published:** 2023-09-05

**Authors:** Shohei Kataoka, Yuta Morioka, Miwa Kanai, Kyoichiro Yazaki, Shun Hasegawa, Satoshi Higuchi, Daigo Yagishita, Morio Shoda, Junichi Yamaguchi

**Affiliations:** ^1^ Department of Cardiology Tokyo Women's Medical University Shinjuku‐ku Tokyo Japan; ^2^ Clinical Research Division for Heart Rhythm Management Department of Cardiology Tokyo Women's Medical University Shinjuku‐ku Tokyo Japan

**Keywords:** cardiac resynchronization therapy defibrillator, heart failure, ventricular arrhythmia

The HeartLogic multisensor algorithm™ (Boston Scientific) calculates the HeartLogic score based on heart sounds, nighttime heart rate, thoracic impedance, respiratory rate, and patient activity level. The MultiSENSE study showed that the HeartLogic multisensor algorithm had a high negative predictive value and good sensitivity for heart failure (HF) events.^1^ However, there were unexplained alerts. The HeartLogic alert state was shown to be associated with atrial fibrillation occurrence.^2^ We hypothesized that HeartLogic scores would be elevated in ventricular arrhythmia (VA) events. Therefore, the present retrospective, single‐center, observational study investigated whether the HeartLogic multisensor algorithm responds to VA events.

Thirty‐five patients who underwent cardiac resynchronization therapy defibrillator (CRT‐D) implantation with the HeartLogic multisensor algorithm at the Tokyo Women's Medical University between 2017 and 2021 were included in this study, which was approved by the Ethics Committee of Tokyo Women's Medical University (No. 4159‐R2).

The HeartLogic score was examined for both VA and HF events, and the prediction accuracy was determined using a cut‐off value of 16, as previously described.^1^ Since it is not possible to define a HeartLogic score without any events, using the receiver operating characteristic curve to calculate the optimal cut‐off value for VA event prediction was unfeasible. Therefore, we compared the HeartLogic scores between VA and HF events. VA events were defined as appropriate CRT‐D therapy, including antitachycardia pacing and defibrillation for ventricular tachycardia (VT) or ventricular fibrillation. The HeartLogic score for VA events was defined as the HeartLogic score on the day of CRT‐D therapy. HF events were defined as HF hospitalization, unscheduled outpatient visits with intravenous treatments or augmented oral HF medications,^1^ death from HF, and left ventricular assist device (LVAD) implantation. The HeartLogic score for HF events was defined as the HeartLogic score on the day of the event. All HF events were confirmed by medical records.

Categorical variables are presented as numbers and proportions and were compared using Fisher's exact test. Continuous variables are presented as mean and standard deviation or median with interquartile range and were compared using the Mann–Whitney U test. HeartLogic scores between VA and HF events were compared using the Wilcoxon signed‐rank test. The time from the onset of increase in the HeartLogic score to the event was also compared in the same way. The onset of an increase in the HeartLogic score was defined as the point at which the HeartLogic score was zero before HF or VA event occurrence. Statistical significance was set at *p*‐values <.05. JMP Pro 16® software (SAS Institute Inc.) was used for analysis.

All 35 patients underwent CRT‐D device implantation. The mean age was 62.7 ± 15.2 years, and 26 (74.3%) patients were male. The most common underlying cardiac diseases were dilated cardiomyopathy (18 patients, 51.4%), followed by hypertrophic cardiomyopathy (6 patients, 17.1%) and ischemic cardiomyopathy (6 patients, 17.1%). The left ventricular ejection fraction was 31.1 ± 8.2%. Indications for defibrillators and tachycardia detection zones are shown in Table 1.

**TABLE 1:** Baseline characteristics.

	Total (*N* = 35)
Age (years)	62.7 ± 15.2
Male	26 (74.3)
Underlying heart disease	
DCM	18 (51.5)
HCM	6 (17.1)
ICM	6 (17.1)
Others	5 (14.3)
LVEF	31.1 ± 8.2
NYHA	
I	3 (8.5)
II	19 (54.3)
III	12 (34.3)
IV	4 (11.4)
Comorbidity	
Hypertension	12 (34.3)
Diabetes	10 (28.6)
Atrial fibrillation	17 (48.6)
Paroxysmal	12 (34.3)
Persistent	5 (14.3)
Implantable device	
CRT‐D	35 (100)
Defibrillator indication	
Primary	29 (82.9)
Secondary	6 (17.1)
Zone settings	
3 zones	13 (37.1)
2 zones	22 (62.9)
Tachycardia detection zone	
VF zone, bpm	217.1 ± 11.0
VT zone, bpm	165.7 ± 12.8
Slow VT zone, bpm	135.0 ± 11.7

Abbreviations: CRT‐D, cardiac resynchronization defibrillator; DCM, dilated cardiomyopathy; HCM, hypertrophic cardiomyopathy; ICM, ischemic cardiomyopathy; LVEF, left ventricular ejection fraction; VF, ventricular fibrillation; VT, ventricular tachycardia.

During a median follow‐up of 1081 (interquartile range [IQR]: 745–1454) days, 104 events, including 80 VA and 24 HF events, were observed in 19 of the 35 patients (54%). The 24 HF events included 13 HF‐related hospitalizations, 9 unscheduled outpatient visits, 1 HF‐related death, and 1 LVAD implantation. From the total number of events (*n* = 104), we calculated the accuracy based on the number of true positive and negative events. The accuracy for predicting HF and VA events was 76.9% and 23.1%, respectively. The HeartLogic scores at the time of the VA and HF events were 8 (IQR: 5–11) and 13 (IQR: 7–19), respectively; the latter was significantly higher than that at the time of the VA events (*p* = .002) (Figure 1). Most HF events were related to mild HF events such as HF‐related hospitalizations and unscheduled outpatient visits. Therefore, we separately analyzed more severe events, including HF‐related death and LVAD implantation. The HeartLogic scores for HF‐related death and LVAD implantation were 43 and 38, respectively. The time from the HeartLogic score increase to VA and HF events was 23 (13–37) days and 31 (20–78) days, respectively. The time from the HeartLogic score increase to the VA event was significantly shorter than that from the HF event to HF‐related hospitalization (Figure 2).

**FIGURE 1:**
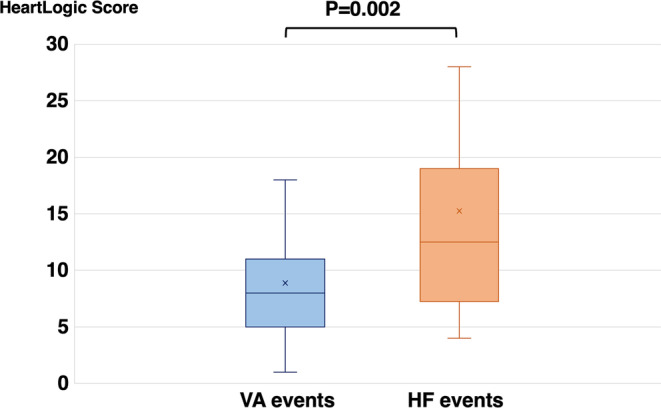
HeartLogic score at events. The HeartLogic scores at the time of ventricular arrhythmia (VA) events and heart failure (HF) events (HF hospitalization, unscheduled outpatient visits with intravenous treatment, HF death, and left ventricular assist device implantation) were 8 (interquartile range: 5–11) and 13 (7–19), respectively. The HeartLogic score of VA events was significantly lower than that of HF events.

**FIGURE 2:**
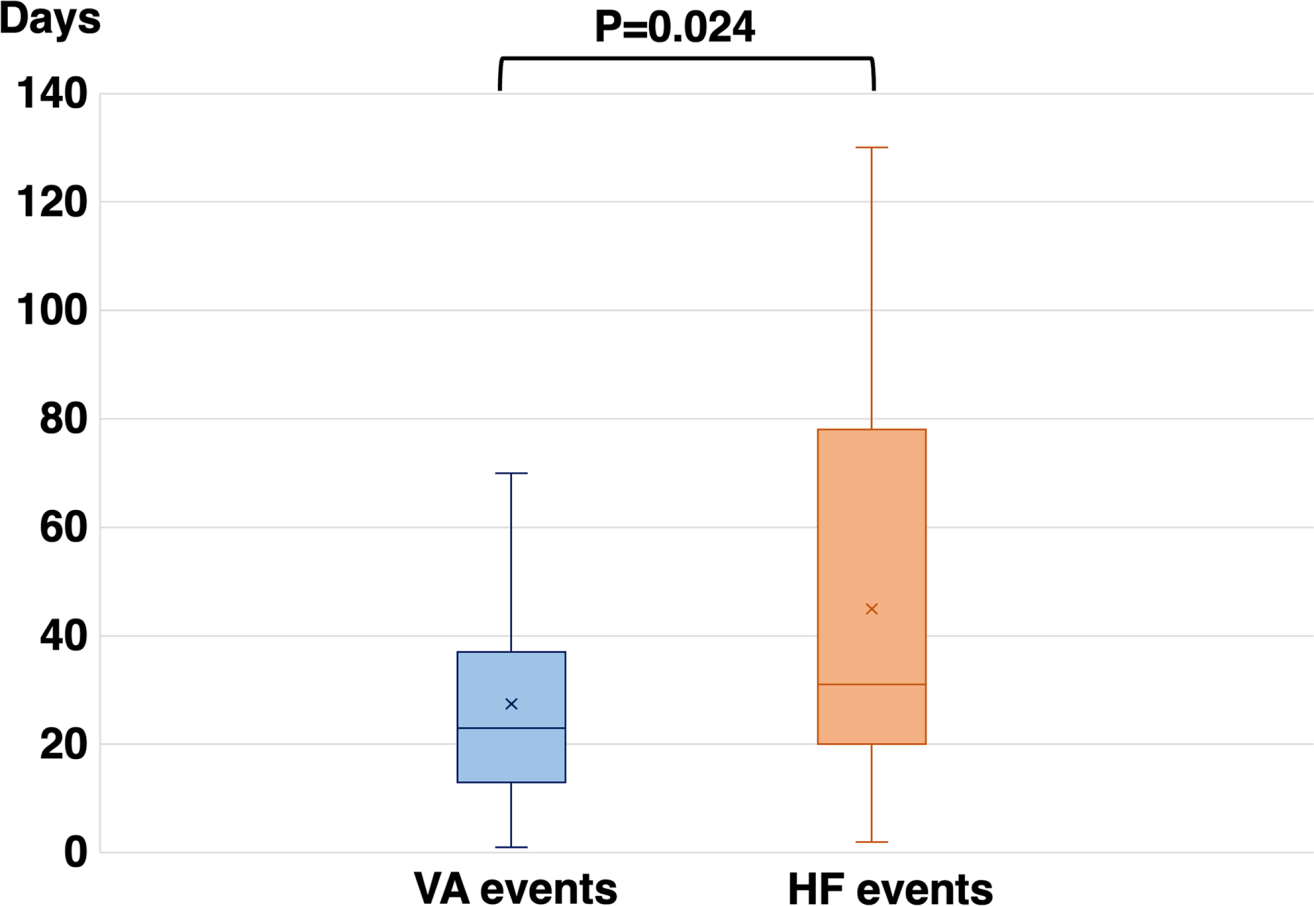
Time from HeartLogic score increase to the event. The time from the HeartLogic score increase to ventricular arrhythmia (VA) events and heart failure (HF) events was 23 (interquartile range: 13–37) days and 31 (20–78) days, respectively. The time from the HeartLogic score increase to VA events was significantly shorter than that to HF events.

In a representative case of ischemic cardiomyopathy of a 70‐year‐old man after CRT‐D implantation, the HeartLogic score increased to approximately 30 while being hospitalized for HF; however, after discharge following treatment with intravenous diuretics, the HeartLogic score decreased to 0. Approximately 2 months after discharge, the HeartLogic score was slightly increased. Seven days later, the patient underwent anti‐tachycardia pacing for VT. The HeartLogic score at that time was 4. Approximately 1 month later, VA events were observed again. Shock therapy was administered following anti‐tachycardia pacing for VT. The HeartLogic score was 9 (Figure 3).

**FIGURE 3:**
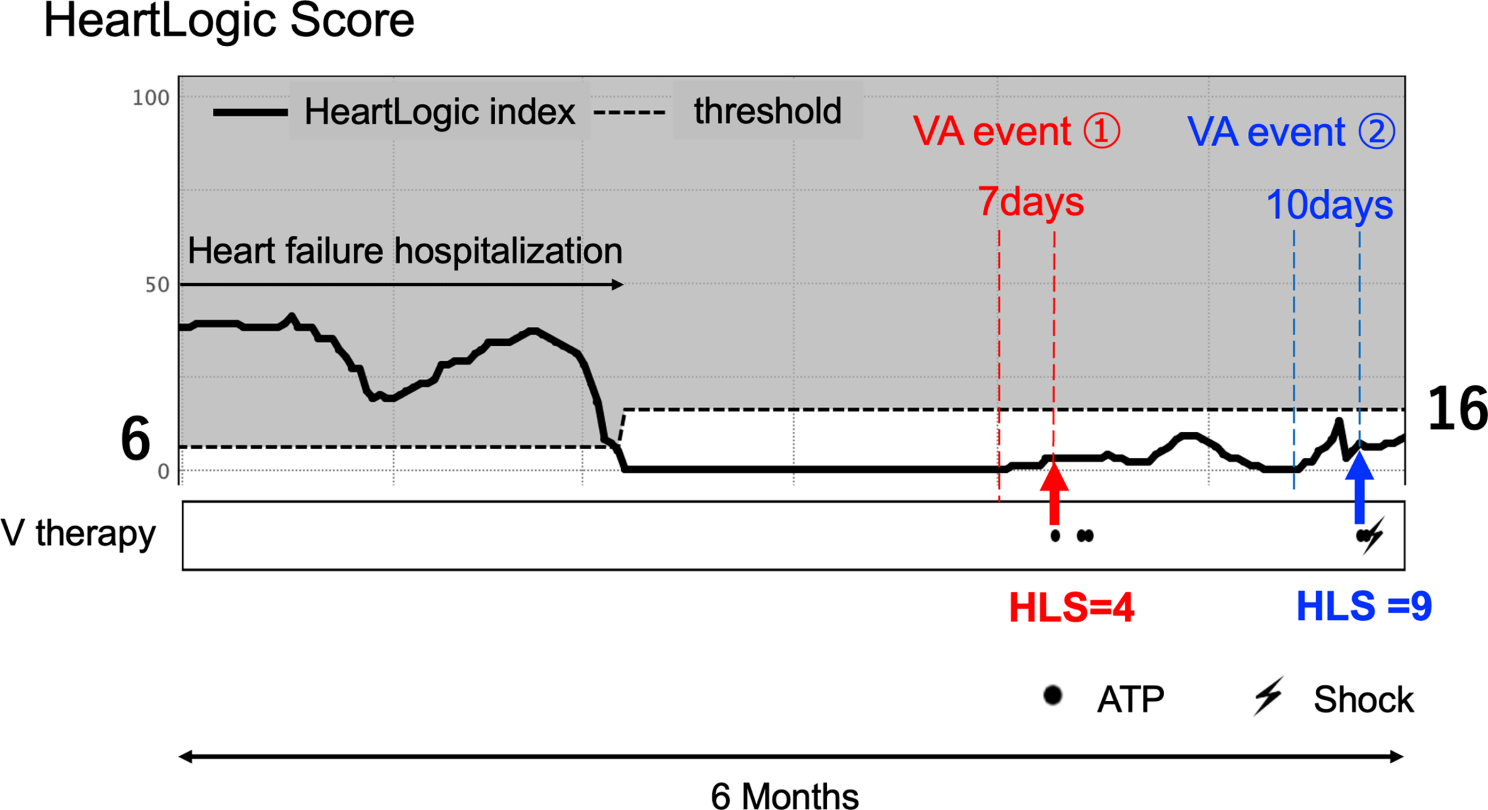
Representative case of ventricular arrhythmia event with an elevated HeartLogic score. We present a representative case of a 70‐year‐old man with ischemic cardiomyopathy after a cardiac resynchronization therapy defibrillator implantation. While hospitalized for heart failure, the HeartLogic score increased to approximately 30; however, after discharge, the HeartLogic score decreased to 0. Two months after discharge, a slight increase in the HeartLogic score was observed. Seven days later, the patient underwent anti‐tachycardia pacing for ventricular tachycardia with a HeartLogic score of 4 at the time of the event. The patient underwent ICD therapy again one month later. In the second event, the HeartLogic score was 9.

The key findings of the present study were: the median HeartLogic score for VA events was 8, and the median time from the HeartLogic score increase to the VA event was 23 days. Therefore, this study demonstrates that the HeartLogic multisensor algorithm responds prior to VA events, as hypothesized.

The HeartLogic score is designed to predict worsening HF. Therefore, the mechanism of VA observed in this study may be related to worsening HF. Increased intracardiac pressure associated with worsening HF causes stretching of ventricular myocytes, prolonging the duration of the action potential and shortening the effective refractory period.^3^ Additionally, neurohumoral factors increase with worsening HF. This sympathetic activation increases norepinephrine and endothelin‐1 release from the heart, which is involved in the development of VAs.^4^ Altered calcium handling is also observed during worsening HF and is involved in the occurrence of triggered premature ventricular contractions due to early after‐depolarizations and delayed after‐depolarizations.^5^ Due to these mechanisms, HF exacerbation induces VAs and contributes to an increase in the substrate of VAs. The present study revealed that the HeartLogic score cut‐off value of 16, originally proposed for predicting HF events in the MultiSENSE study, was suboptimal for predicting VA events. Therefore, in large‐scale future studies, the optimal cut‐off value for the HeartLogic score for predicting VA must be determined.

In the present study, the median HeartLogic score at the time of the HF events was 13, which was slightly lower than the optimal cut‐off value in the MultiSENSE study.^1^ The different thresholds for hospital visits and HF‐related hospitalization between Japan and the United States may be attributable to this variation. In Japan, access to hospitals is easier than that in the US, allowing patients to present to hospitals with relatively mild symptoms of worsening HF. This may affect the HeartLogic score at the time of an HF event. The threshold for hospitalization may also be lower in Japan. In addition, approximately 35% of HF events were unscheduled outpatient visits with intravenous treatment, which may have influenced the lower HeartLogic scores of HF events in this study.

This study had two major limitations. First, it was a single‐center, retrospective study with a relatively small sample size, potentially causing selection bias. Second, the small number of HF events may have led to underestimation of the HeartLogic score at the time of HF events, given that 35% of HF events were unscheduled outpatient visits due to mild HF exacerbation.

In conclusion, the HeartLogic multisensor algorithm responded prior to VA events, possibly because HF exacerbations were closely associated with VA occurrence. Thus, the HeartLogic Score may help predict VA events in some patients treated by CRT‐D, especially after determining the optimal cut‐off value.

## FUNDING INFORMATION

None.

## CONFLICT OF INTEREST STATEMENT

The authors have no competing interests to disclose.

## PATIENT CONSENT STATEMENT

Written informed consent was obtained from all patients.

## ETHICS APPROVAL STATEMENT

This study was approved by the Ethics Committee of Tokyo Women's Medical University (No. 4159‐R2).

## CLINICAL TRIAL REGISTRATION

Not applicable.

## Data Availability

The deidentified participant data will not be shared.
